# Supplementary material for: Two-site H2O2 photo-oxidation on haematite photoanodes
Source: Nat Commun. 2018 Oct 9;9:4060. doi: 10.1038/s41467-018-06141-0 (PMC6177486; doi:10.1038/s41467-018-06141-0)
Supplement: Supplementary file 1 — Supplementary Information [file 41467_2018_6141_MOESM1_ESM.pdf]

Supplementary Information

A Tale of Two Sites in H_2O_2 Photo-Oxidation on Hematite Photoanodes

Yotam Y. Avital, Hen Dotan, Dino Klotz, Daniel A. Grave, Anton Tsyganok, Bhavana Gupta, Sofia Kolusheva, Iris Visoly-Fisher, Avner Rothschild, and Arik Yochelis

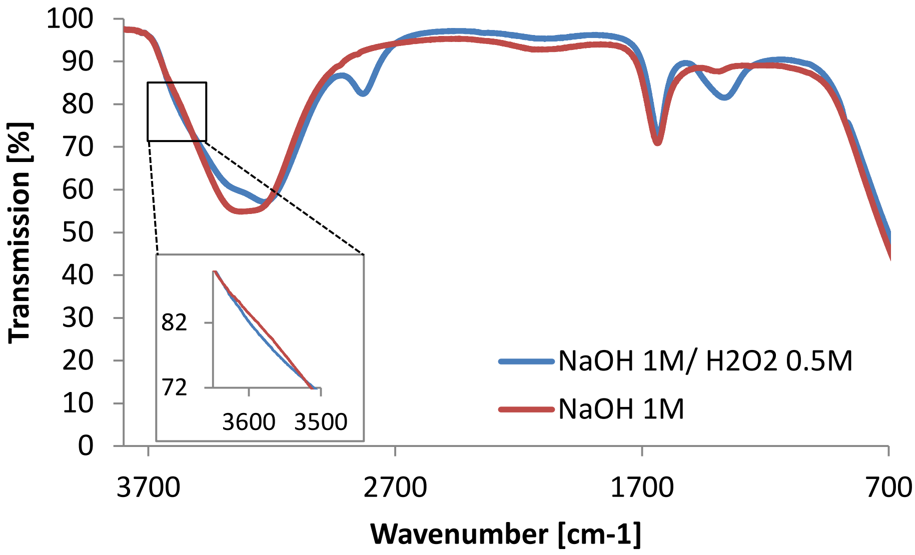

a

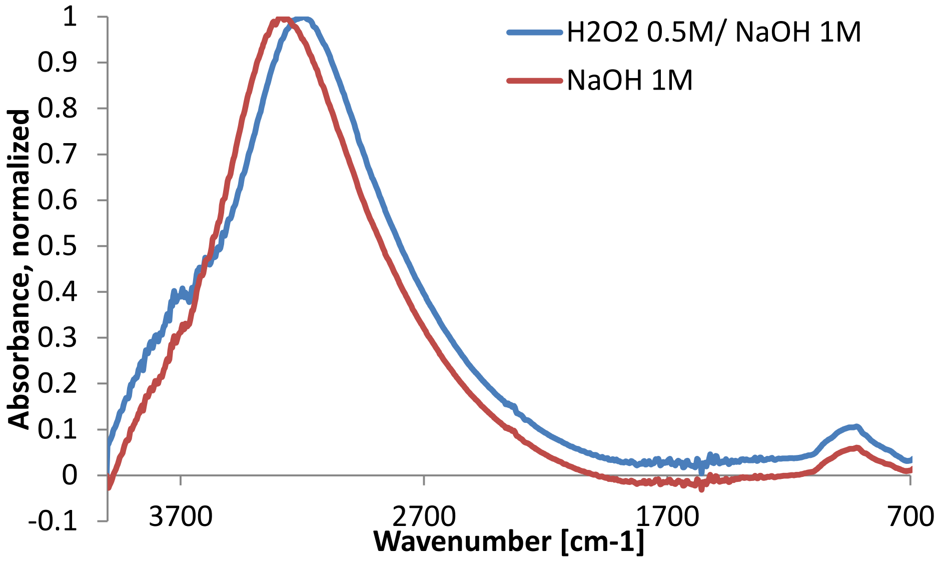

b

Supplementary Figure 1: FTIR spectra. (a) Electrolyte solutions and (b) hematite photoanodes after soaking in the respective electrolyte solutions (normalized), as indicated in the legend.

Supplementary methods: Spectroscopic measurements

Fourier transform infrared spectroscopy (FTIR) was used to detect the adsorption of OOH^{-} species to Fe-OH, as suggested in eq. 7. Supplementary Figure 1(a) shows spectra of the NaOH 1M electrolyte with and without 0.5M H_2O_2, The most prominent differences upon H_2O_2 addition are additional peaks at 1360 and 2830 cm^{-1} and a decrease in the 3330 cm^{-1} peak intensity, in agreement with previously published results.[1] A very small additional shoulder is observed at 3550 cm^{-1} (inset in Supplementary Figure 1(a)). We therefore looked for these differences in FTIR spectra of hematite photoanodes soaked in the respective solutions. Supplementary Figure 1(b) shows these spectra, with the expected Fe-OH peak near 920 cm^{-1} and a very broad OH-related peak around 3200 cm^{-1}, typical of hematite spectra.[2,3] The main differences between the spectra are a shift of the OH-peak from 3290 cm^{-1} after soaking in NaOH electrolyte to 3200 cm^{-1} after soaking in H_2O_2 containing electrolyte, and a shoulder between 3600-3690 cm^{-1} in the latter. We postulate that both are related to H_2O_2 adsorption, as the decreased 3330 cm^{-1} peak intensity and the additional 2830 cm^{-1} peak, observed in the H_2O_2-containing electrolyte's spectrum (Supplementary Figure 1(a)), can induce the OH-peak shift to smaller wavenumbers in the hematite spectrum. The additional small shoulder is related to that observed in the solution spectra (inset in Supplementary Figure 1(a)).

Supplementary References

1. Dotan, H. *et al.* Resonant light trapping in ultrathin films for water splitting. *Nat. Mater.* **12,** 158–164 (2013).
2. Kim, J. Y. *et al.* Single-crystalline, wormlike hematite photoanodes for efficient solar water splitting. *Sci. Rep.* **3,** 2681 (2013).
3. Guo, X., Wang, L. & Tan, Y. Hematite nanorods Co-doped with Ru cations with different valence states as high performance photoanodes for water splitting. *Nano Energy* **16,** 320–328 (2015).
